# Seeking homeostasis: temporal trends in respiration, oxidation, and calcium in SOD1 G93A Amyotrophic Lateral Sclerosis mice

**DOI:** 10.3389/fncel.2015.00248

**Published:** 2015-07-01

**Authors:** Cameron W. Irvin, Renaid B. Kim, Cassie S. Mitchell

**Affiliations:** Department of Biomedical Engineering, Georgia Institute of Technology – Emory University, Atlanta, GAUSA

**Keywords:** ALS, motor neuron disease, mitochondria, oxidative stress, Ca^2+^, energy metabolism

## Abstract

Impairments in mitochondria, oxidative regulation, and calcium homeostasis have been well documented in numerous Amyotrophic Lateral Sclerosis (ALS) experimental models, especially in the superoxide dismutase 1 glycine 93 to alanine (SOD1 G93A) transgenic mouse. However, the timing of these deficiencies has been debatable. In a systematic review of 45 articles, we examine experimental measurements of cellular respiration, mitochondrial mechanisms, oxidative markers, and calcium regulation. We evaluate the quantitative magnitude and statistical temporal trend of these aggregated assessments in high transgene copy SOD1 G93A mice compared to wild type mice. Analysis of overall trends reveals cellular respiration, intracellular adenosine triphosphate, and corresponding mitochondrial elements (Cox, cytochrome c, complex I, enzyme activity) are depressed for the entire lifespan of the SOD1 G93A mouse. Oxidant markers (H_2_O_2_, 8OH2′dG, MDA) are initially similar to wild type but are double that of wild type by the time of symptom onset despite early post-natal elevation of protective heat shock proteins. All aspects of calcium regulation show early disturbances, although a notable and likely compensatory convergence to near wild type levels appears to occur between 40 and 80 days (pre-onset), followed by a post-onset elevation in intracellular calcium. The identified temporal trends and compensatory fluctuations provide evidence that the “cause” of ALS may lay within failed homeostatic regulation, itself, rather than any one particular perturbing event or cellular mechanism. We discuss the vulnerabilities of motoneurons to regulatory instability and possible hypotheses regarding failed regulation and its potential treatment in ALS.

## Introduction

Amyotrophic Lateral Sclerosis (ALS) is a late-onset neurodegenerative disease consisting of progressive muscle atrophy, muscle paralysis, dysarthria, dysphagia, and dyspnea. While there has been much research conducted on the disease, the precise causes and effective treatments have remained elusive. Transgenic mice, and namely the superoxide dismutase 1 glycine 93 to alanine mutation (SOD1 G93A), have served as the predominant means by which to investigate the underlying cellular pathophysiology (Pfohl et al., 2015). A multitude of categorical disturbances have been identified, which are described in detail in a recent informatics-based systematic review of the SOD1 G93A field (Kim et al., 2015): apoptosis, including changes in pro- and anti-apoptotic signals; energetics, including mitochondrial dysfunction, adenosine triphosphate (ATP) depletion, and calcium homeostasis; excitability, including hypoexcitability, hyperexcitability, and excitotoxicity; genetic transcription, including damage to mRNA and DNA; inflammation, due to reactive microglia and astrocytes; oxidative stress, from the production of free radicals; proteomics, including the build-up of mutant protein aggregates and reduced autophagy or proteasome function; and systemic function, which also includes potential non-neuromuscular contributors.

This Frontiers Research Topic and present study focuses on a unique and highly inter-related triad of the ALS pathophysiology: the role of mitochondria, oxidative stress, and altered calcium homeostasis. Most of the previous experimental work has focused on identifying the presence of impairments in this triad using a wide variety of specific methods and measures, such as recording of the mitochondrial potential, evaluation of intracellular ATP concentration, electrophysiological assessment of calcium entry, and measurement of intracellular free radicals. While the presence of deficiencies in mitochondria, oxidative regulation, and calcium homeostasis has been well established, their timing, as a function of ALS disease initiation and progression, is less understood. The goal of this work is to evaluate their overall temporal trends, pre-natal through death, in the SOD1 G93A transgenic ALS mouse model. In this systematic review of 45 articles, we aggregate *in vitro*, embryonic, and *in vivo* experimental measurements of cellular respiration, mitochondrial mechanisms, oxidative and anti-oxidative markers, and intracellular calcium in both SOD1 G93A transgenic ALS mice and in wild type control mice. We evaluate the magnitude and statistical trend of these assessments in SOD1 G93A mice compared to wild type mice and examine changes over the course of the SOD1 G93A mouse life span.

## Materials and Methods

The general method includes: (1) identifying and recapturing published experimental data for SOD1 G93A transgenic mouse and wild type control mouse assessments of mitochondrial function, oxidative stress, and calcium homeostasis; (2) Normalizing recaptured data to temporally compare the assessed measures; (3) Performing a Gaussian average to temporally interpolate the values and develop a visual trend line for each measure and category of measures.

### Inclusion and Exclusion Criteria

Potential articles were identified under the PubMed search criteria of (G93A OR transgenic mouse) AND (ALS OR “Amyotrophic Lateral Sclerosis”). Initial exclusion criteria consisted of: non-English language articles; articles for which full-text pdf downloads were unavailable (see Data Recapture); and articles labeled as literature reviews.

Keyword searches of recaptured figure captions and within figure text were performed to find relevant articles from the initial literature pool (see Data Recapture) using the following terms: calcium and its permutations, including Ca^2+^, Ca, etc.; mitochondria and its permutations (mito*); oxidative stress and its permutations (oxid*), reactive oxygen species (ROS), free radicals, hydrogen peroxide (H_2_O_2_), nitric oxide (NO), malondialdehyde (MDA), 8-hydroxy-2′-deoxyguanosine (8OH2′dG), heat shock proteins (HSPs). Study-specific inclusion criteria required the use of both non-treated SOD1 G93A and wild type transgenic mice for a given quantitative experimental measurement. “Non-treated” consisted of controlled experimental assessments of the measured parameter(s) without the application of chemicals or processes meant to intentionally attempt to modify the assessed measures or related physiology.

### Data Recapture

Articles were either downloaded using PubMed Central or from e-journal subscriptions available from the libraries of Georgia Institute of Technology and Emory University. Data was recaptured from the following article locations (Kim et al., 2015), referred to as entities: article title; abstract; figure captions; within figure text (*x*–*y* axis labels, bar graph categorical labels, legends, etc.); and data series and response values (e.g., quantifiable figure/table data). Data was scraped from the full-text pdf article. Every data point was reviewed by an independent quality control team to insure complete accuracy.

### Categorical Definitions

Given that each study utilizes its own specific measures, aggregation is a requirement in order to obtain a meaningful visualization of categorical trends and meta-analysis results. Experimental measures were aggregated into four main categories: calcium regulation, cellular respiration, mitochondrial mechanisms, and oxidative regulation. Sub-categories of measures are further defined within each category as discussed below.

#### Calcium Regulation

The calcium regulation category includes experimental assessments of intracellular calcium dynamics, including calcium entry, calcium sinks, free calcium concentration, and calcium sensitivity. Calcium entry encompassed electrophysiological measurements of extracellular calcium entering through the membrane of motoneuron cells (e.g., Ca^2+^ persistent inward current, Ca^2+^ amplitude, Cell Voltage, Cellular Calcium, etc.). Calcium sensitivity included measures of mitochondrial membrane sensitivity to a discretely measured calcium challenge. Calcium sink values encompassed measures of calcium buffering or calcium capacity. Calcium concentration encompassed general cytosolic free calcium levels.

#### Cellular Respiration

The cellular respiration category includes measures of general respiration rate as well as intracellular concentrations of ATP and adenosine diphosphate (ADP). Measures of the general respiration rate include production of heme, oxygen consumption, or respiration control ratio.

#### Respiration Mechanisms

Respiration mechanisms included experimental assessment of complex I activity, COX activity, Cytochrome C levels, and general mitochondrial enzyme activity (including Complex III, IV, V). These parameters are involved the electron transport chain (ETC), which leads to the production of ATP.

#### Oxidative Regulation

Experimentally assessed contributors to oxidative stress include hydrogen peroxide (H_2_O_2_) production and the oxidant markers MDA and 8-hydroxy-2′-deoxyguanosine (8OH2′dG). The concentration of HSP was also incorporated in the oxidative regulation category given their neuroprotective effects and potential impact on the slowing of oxidant-induced symptoms. Specific HSPs included HSP 25, 27, and 70.

### Tissue Sources

*In vivo* tissues primarily consisted of SOD1 G93A mouse brain or spinal cord cells; in fact, 35 articles identified at least one of these two regions as a source. Some articles utilized homogenized tissue or multiple tissue sources (Leclerc et al., 2001; Damiano et al., 2006; Johnson et al., 2011; Miana-Mena et al., 2011), which included spinal cord or brain tissue with other systemic tissues, such as blood, liver, soleus, diaphragm, and liver. Tissues used for *in vitro* assessment were more varied. Cell lines mostly included standard SOD1 G93A transfected mice cells. However, other G93A-transfected sources included SH-SY5Y cells (Carri et al., 1997; Goos et al., 2007; Pesaresi et al., 2011), NSC-34 cells (Liu et al., 2002; Ferri et al., 2008; Mali and Zisapel, 2009; Crippa et al., 2010), yeast (Gunther et al., 2004; Kloppel et al., 2010), and bacteria (Singh et al., 1998).

### Analysis

The ratio of SOD1 G93A to wild type (e.g., SOD1 G93A/wild type) is used to normalize each assessed measure. Each study was normalized to its own published wild type data. For each included measure or category of measures, the ratio of SOD1 G93A to wild type is plotted versus time. Data from each article is given equal weight. For ease of visualization, all *in vitro* cell line experimental data is plotted as -20 days and embryonic experimental data is plotted as -5 days; *in vivo* data is plotted at its corresponding post-natal day of experimental assessment. A standard Gaussian average was performed to interpolate values in-between the raw experimental data points and to produce trend lines indicative of the general aggregate behavior of each experimentally measured parameter.

## Results

In total, 262 data points from 45 unique papers were collected and normalized for inclusion in this meta-analysis. **Table 1** shows the experimental data point and article distribution to each of the defined categories and subcategories of measures. The category, mitochondrial mechanisms, which contains measurements of constituents necessary for cellular respiration, includes 67 values from 10 unique articles. Cellular respiration, which includes the assessed respiration rate and intracellular concentrations of ATP and ADP, includes 59 values from 12 unique articles. The oxidative stress category, which includes oxidative markers and anti-oxidative HSPs, has a total of 73 values from 14 unique articles. Intracellular calcium, which contains measures examining intracellular calcium homeostasis, contains 63 data points from 15 unique articles.

**Table 1:** Categorization, distribution, and sources of included experimental data.

	Papers	Values	Sources
**Calcium regulation**			
Entry	2	12	Johnson et al. (2011), Quinlan et al. (2011)
Sensitivity	2	11	Pieri et al. (2003), Damiano et al. (2006)
Sink	6	14	Pieri et al. (2003), Damiano et al. (2006), Igoudjil et al. (2011), Milanese et al. (2011), Pesaresi et al. (2011), Tradewell et al. (2011)
Concentration	9	26	Carri et al. (1997), Kruman et al. (1999), Beers et al. (2001), Goos et al. (2007), Guatteo et al. (2007), Milanese et al. (2011), Nutini et al. (2011), Panov et al. (2011), Tradewell et al. (2011)
**Mitochondrial mechanisms**			
Complex I	1	3	Loizzo et al. (2010)
Enzyme activity	5	49	Leclerc et al. (2001), Jung et al. (2002), Mattiazzi et al. (2002), Wendt et al. (2002), Gunther et al. (2004)
COX	1	4	Kirkinezos et al. (2005)
Cytochrome C	5	11	Guégan et al. (2001), Jung et al. (2002), Liu et al. (2002), Kirkinezos et al. (2005), Damiano et al. (2006)


**Cellular respiration**			
Adenosine triphosphate (ATP)	4	21	Browne et al. (2006), Ferri et al. (2008), Mali and Zisapel (2009), Peixoto et al. (2013)
Adenosine diphosphate (ADP)	2	18	Leclerc et al. (2001), Browne et al. (2006)
Respiration rate	7	20	Gunther et al. (2004), Damiano et al. (2006), Cassina et al. (2008), Igoudjil et al. (2011), Panov et al. (2011), Miquel et al. (2012), Zhao et al. (2012)
**Oxidative Regulation**			
8-hydroxy-2′-deoxyguanosine, 8OH2′dG	1	4	Fang et al. (2010)
Hydrogen peroxide, H_2_O_2_	5	7	Singh et al. (1998), Liu et al. (1999, 2009), Takamiya et al. (2003), Kloppel et al. (2010)
Malondialdehyde (MDA), C_3_H_4_O_2_	5	36	Hall et al. (1998), Liu et al. (1999), Fang et al. (2010), Miana-Mena et al. (2011), Seo et al. (2011)
Heat shock proteins (HSP)	5	26	Vleminckx et al. (2002), Batulan et al. (2003), Maatkamp et al. (2004), Jaarsma et al. (2008), Crippa et al. (2010)

*There are four major categories of measures: intracellular calcium, mitochondrial mechanisms, cellular respiration, and oxidative regulation). Sub-categories of measures, as defined in the Methods, are listed under each category along with the number of included unique articles and extracted data points. Each data point represents a paired SOD1 G93A and wild type mouse assessment at a discrete time point.*

### Cellular Respiration is Depressed for Entire Lifespan

**FIGURE 1:**
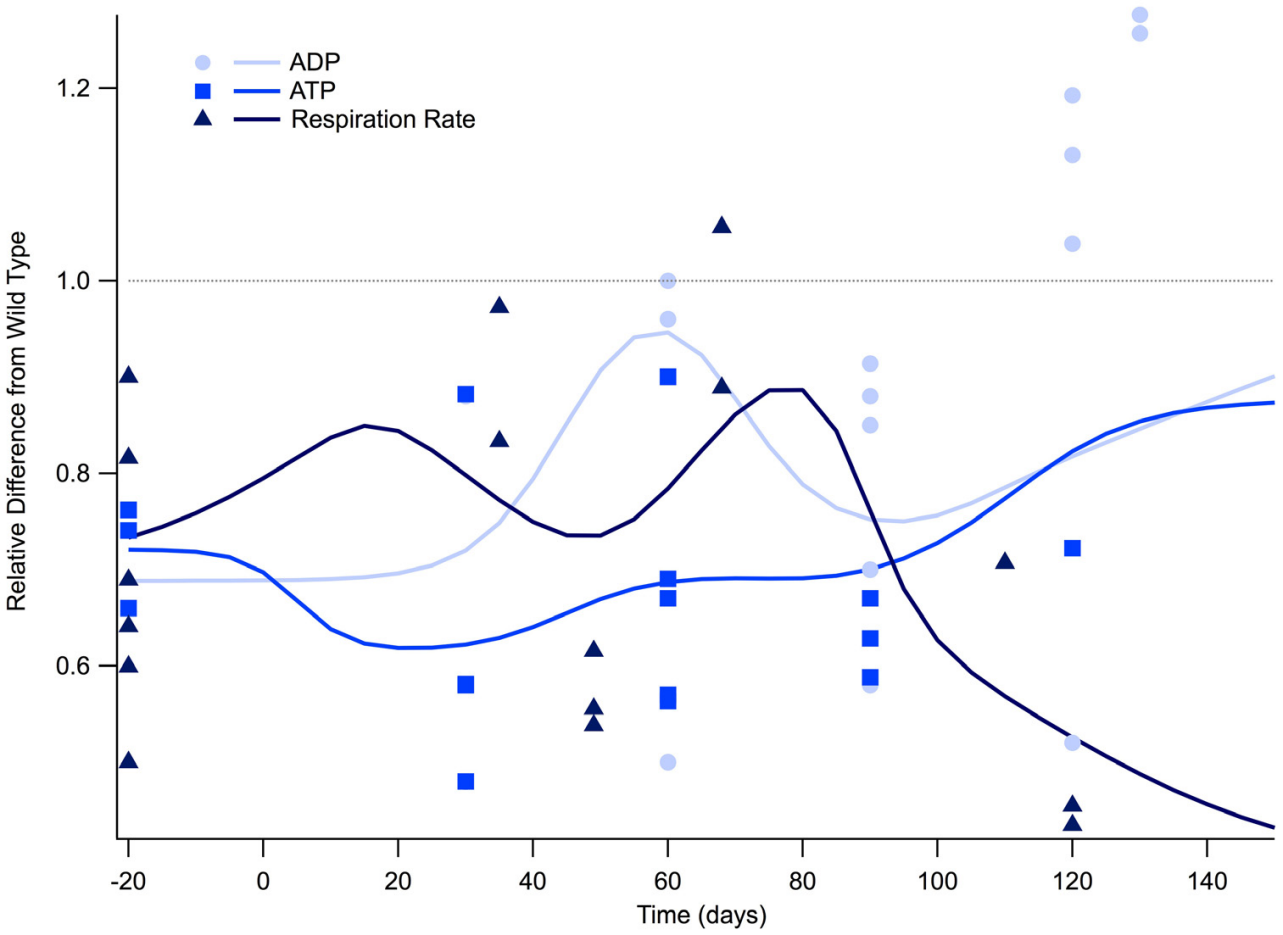
**Cellular respiration is depressed for the lifespan of the SOD1 G93A transgenic mouse model**. The ratio of SOD1 G93A to wild type (SOD1 G93A/wild type) is plotted over time for experimental measures of adenosine triphosphate (ATP), adenosine diphosphate (ADP), and general respiration rate. Solid lines illustrate predicted trend lines. For visualization purposes, *in vitro* data is plotted at -20 days and *in vivo* time points are plotted at their corresponding post-natal day of assessment, 0–150 days. A gray dotted line is provided to show the expected wild type or homeostatic value. Trend lines are generated based on a Gaussian average of the normalized data points.

Among the most interesting trends found in this meta-analysis study is that ATP production, along with general respiration rates, were found to be depressed for the entire lifespan of the SOD1 G93A mouse (**Figure 1**). While it has been well documented that respiration rates are lowered in ALS (Kawamata and Manfredi, 2010; Cozzolino and Carri, 2012; Peixoto et al., 2013) even well before physical pathologies develop (Browne et al., 2006), this meta-analysis supports the assertion that this phenomenon is a trend that, at least in the high-copy SOD1 G93A transgenic mouse model, is present since birth. That is, the SOD1 G93A mice have notable depression of cellular energetics well before symptom onset, and this depression remains throughout the course of disease progression.

**Figure 1** illustrates the ratio of transgenic mouse to wild type mouse experimentally measured levels of intracellular ATP, ADP, and respiration rate. All of the *in vitro* cellular data (plotted as -20 days in **Figure 1**) collected for intracellular ATP concentration and respiration rate fall well below their wild type counterparts. Examining the mathematical mean of *in vitro* measures of ATP and respiration rate reveal that SOD1 G93A levels are approximately 70% of those seen in wild type, which is equivalent to a 30% reduction.

Post-natal *in vivo* assessment of intracellular ATP and respiration rate in SOD1 G93A mice also shows substantial depression compared to wild type mice. While ATP and respiration rate is depressed throughout the life span of the SOD1 G93A mouse, there appears to be small fluctuations throughout the disease course. However, more data is necessary to determine whether these small fluctuations are statistically or mechanistically meaningful. ATP is at its lowest at the disease end point, where ATP levels approach only 30% of wild type (**Figure 1**). Interestingly, the temporal trend of ADP is slightly different than ATP and respiration rate. For most of the disease course, ADP is depressed in a similar manner to ATP and respiration. However, ADP levels in SOD1 G93A mice show an interesting rise above wild type control mice that occurs near the disease end point. This near-death rise in ADP could be attributed to the cells’ inability to convert ADP to ATP, which would leave ADP in excess. In fact, this same trend in the lowering of the ATP/ADP ratio is seen in clinical patients (Ghiasi et al., 2012).

### Depression of Mitochondrial Mechanisms

**FIGURE 2:**
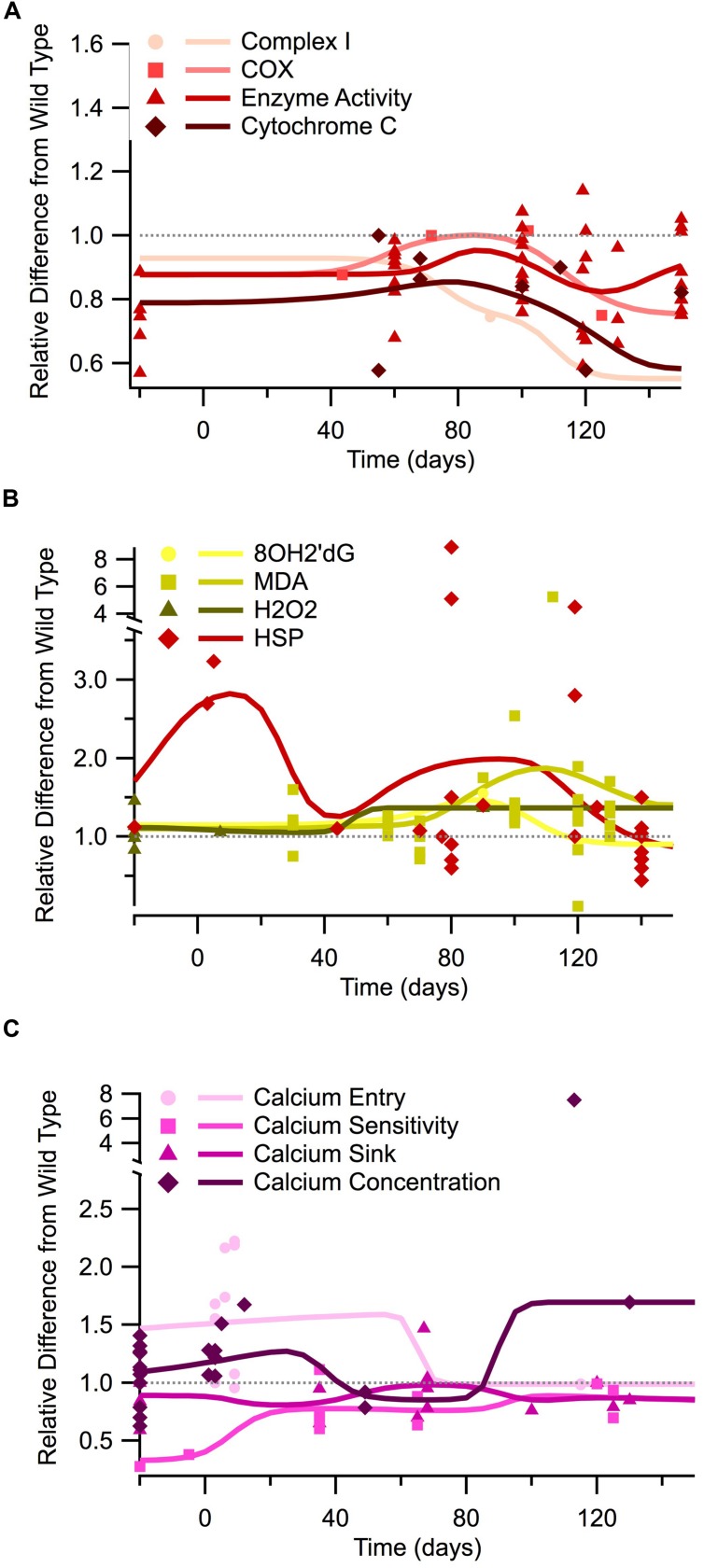
**Temporal trends of mitochondrial mechanisms, oxidant regulation, and calcium regulation in the SOD1 G93A transgenic mouse model.** The ratio of SOD1 G93A to wild type (SOD1 G93A/wild type) is plotted over time for each experimental measure. For visualization purposes, *in vitro* data is plotted at -20 days, embryonic data is plotted at -5 days, and *in vivo* time points are plotted at their corresponding post-natal day of assessment, 0–150 days. A gray dotted line is provided to show the expected wild type or homeostatic value. Trend lines are generated based on a Gaussian average of the normalized data points. **(A)** Mitochondrial mechanisms (Complex I, COX, enzyme activity, and cytochrome c). **(B)** Oxidant markers (8OH2′dG, MDA, H_2_O_2_) and protective heat shock proteins (HSPs). **(C)** Calcium regulation (entry, sensitivity, sink, and cytosolic concentration).

Additionally, measures of mitochondrial mechanisms and signals necessary for respiration and ATP production are similarly depressed throughout the course of the disease. **Figure 2A** illustrates the temporal trends of four different mitochondrial mechanism measurements and signals necessary for cellular respiration: complex I, COX, general enzyme activity, and cytochrome C. *In vitro* measures were only obtainable for enzyme activity, which shows a depression similar to that seen in ATP levels. Post-natal *in vivo* assessment of SOD1 G93A mice reveals that all four of the mitochondrial mechanism measurements are generally depressed compared to wild type. The *in vivo* depression is present at birth and throughout the entire disease duration. Although the mitochondrial mechanism experimental measures remain below wild type, the Gaussian average trend lines identify a potential small bump in mitochondrial mechanism activity near disease onset (around 80 days), which could represent a regulatory compensation mechanism; a larger sample size is necessary to determine if this small bump has possible statistical or mechanistic implications in disease progression. Finally, when the mitochondria’s ability to produce ATP is impaired, there is a compensatory increase in mitochondrial enzyme complexes, especially Complex II, III, IV (Nalbandian et al., 2015). This upward trend in mitochondrial enzymes is seen **Figure 2A** near post-onset and the disease end point.

### Elevation of Oxidants Near Onset

Oxidant levels have been documented as being elevated throughout different stages of ALS (Liu et al., 2002; Mattiazzi et al., 2002; Martin et al., 2007), including pre-onset (around 40 days), onset (80 days), and especially end-stage (120+ days). In **Figure 2B** we plot the temporal trends of three commonly measured oxidants in SOD1 G93A mice (8OH2′dG, MDA, and H_2_O_2_). The data presented in **Figure 2B** reveals that SOD1 G93A intracellular oxidant levels are initially similar to slightly above wild type at birth. However, by pre-onset, levels are mildly elevated, and at onset and end point, oxidant levels are substantially increased compared to wild type. *In vitro* assessment of oxidants does not reveal as pronounced of elevation as the *in vivo* assessments. *In vivo* assessment of oxidants reveals a 1.5 factor increase in oxidants compared to wild type around symptom onset (80 days). *In vivo* assessment near the SOD1 G93A disease end point (120+ days) reveals oxidant levels that are a factor of 2–8 times greater than seen in wild type control mice.

Heat shock proteins, which have an anti-oxidative effect, are also plotted in **Figure 2B**. HSPs in SOD1 G93A mice were found to initially be substantially greater than wild type levels, but they exhibited a fluctuating decline as the disease progressed. However, there appears to be a recurrent delayed rise in HSP as the disease enters the symptomatic stage (around 80 days). This fluctuation has been previously described. It is hypothesized that HSP levels are insufficient to quell the oxidant rise. Thus, decreased HSP levels actually precede motor neuron loss in ALS (Maatkamp et al., 2004).

### Fluctuations of Intracellular Calcium

Calcium homeostasis is critical for both functional neural excitability and normal cellular signaling. There are four main experimental types of calcium regulatory measurements: calcium entry (incoming calcium through ion channels), calcium sensitivity (measurement of the cell’s rate of response to calcium), calcium sink (binding and storage of intracellular calcium, including buffers, transporters, and intracellular stores), and the actual calcium concentration (free intracellular concentration). Each of these measures contributes to the balance or homeostasis of intracellular calcium.

*In vitro* data examining free intracellular calcium concentration is conflicting, with about half of the data points showing elevated calcium and half showing lower intracellular calcium compared to wild type (points plotted at -20 days in **Figure 2C**). These apparent conflicts in *in vitro* intracellular calcium concentration could possibly be explained by the usage of different tissue types for *in vitro* assessment (see Tissue Sources). Measurements of *in vitro* calcium sinks are depressed compared to wild type, ranging from 60 to 80% of that seen in wild type mice, and calcium sensitivity is about 30% of wild type. Post-natal *in vivo* assessment of intracellular calcium and calcium entry at birth reveals levels that are substantially above wild type. However, free calcium and calcium entry appears to dip to near-normal levels during pre-onset. There is limited data for SOD1 G93A mice calcium entry and concentration from onset through end point, but available data revels that calcium appears to rise sharply after onset, resulting in a disease end point intracellular calcium concentration that is a factor 1.5 greater than wild type. *In vivo* assessment of calcium sinks and calcium sensitivity in SOD1 G93A mice show depressed levels compared to wild type from birth through the disease end point. Intuitively, the point at which the calcium sink trend line is highest coincides with the time points when calcium concentration, or free-floating calcium, is lowest.

## Discussion

The results of our systematic review and meta-analysis of 45 articles shed new light on the temporal trends of cellular respiration, oxidative markers, mitochondrial mechanisms, and calcium regulation in the SOD1 G93A transgenic ALS mouse model. By aggregating data, we show that cellular respiration and corresponding mitochondrial mechanism are impaired for the entire lifespan of the SOD1 G93A mouse. Oxidant markers are initially similar to wild type but are more than double that of wild type by the time of symptom onset despite early post-natal elevation of protective HSPs. All aspects of calcium regulation show early disturbances, although a notable and likely compensatory convergence to near wild type levels occurs between 40 and 80 days, which is followed by a divergence after symptom onset.

This systematic review clearly shows that SOD1 G93A mice exhibit a long-term metabolic deficit, however, these symptoms are also present in other ALS mouse models. Dupuis et al. (2004) performed metabolic experiments on both G93A and G86R mice to demonstrate the similarities in mitochondrial function. G37R mice also show significant reduction in ATP production (Coussee et al., 2011). Finally, substantial metabolic disturbances have also been documented in non-SOD transgenic mice, including mice with mutations in TDP-43, FUS, VCP, among others (Carri et al., 2015).

### Interactions within the Respiration-Oxidation-Calcium Triad

**FIGURE 3:**
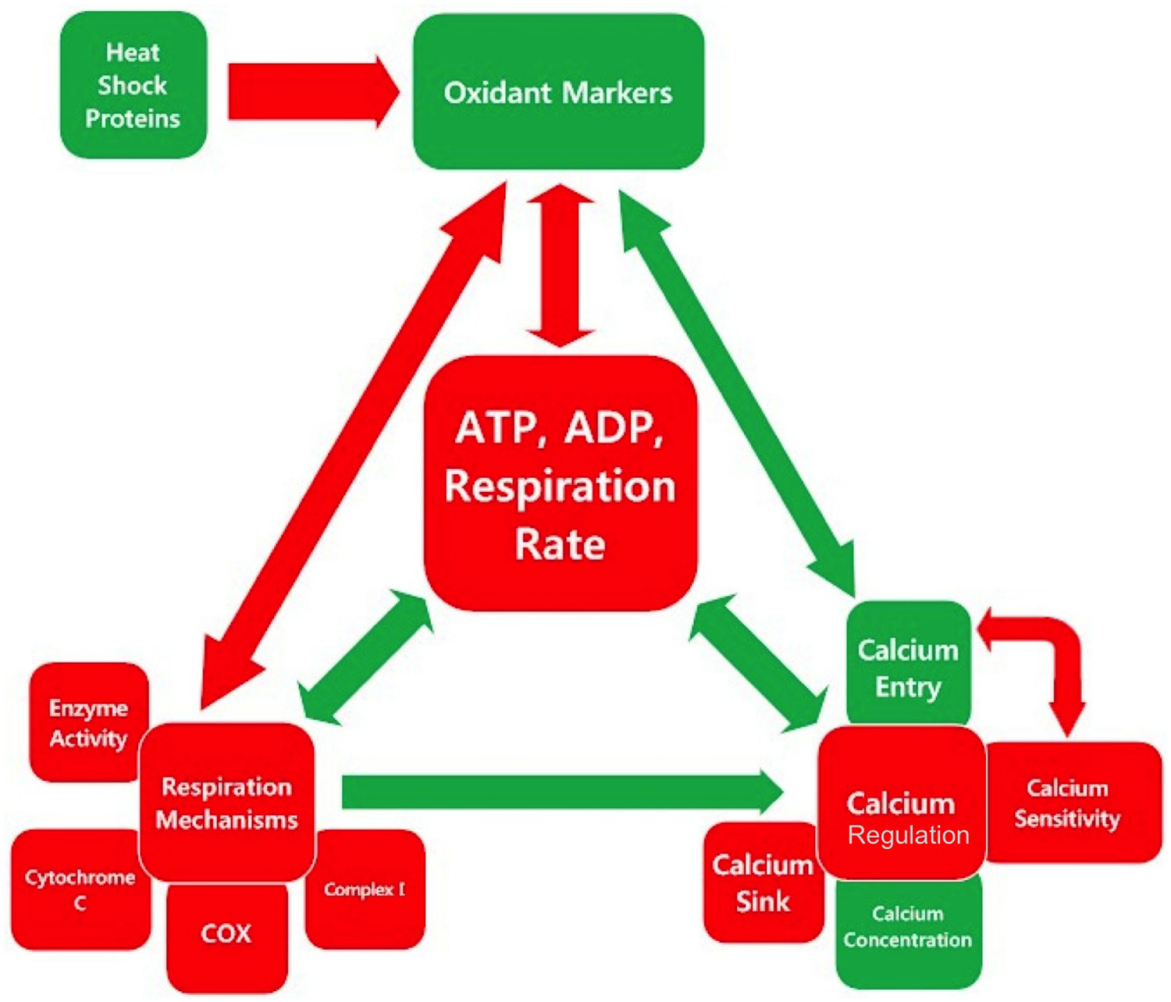
**Inter-relationships in the mitochondrial mechanism-oxidative regulation-calcium regulation triad center around cellular respiration**. Each square is colored based on whether the assessed category is, on average, higher (green), or lower (red) in SOD1 G93A mice compared to wild type as determined by the overall trend line directionality from 0 to 150 days. The color of the arrow indicates the sign of the relationship, and the size indicates the relative magnitude.

There are multiple feedback loops between the triad of cellular respiration, calcium regulation, and oxidative regulation. The inter-relationships between the categories and sub-categories of measurements examined in this meta-analysis are illustrated in **Figure 3**. Red boxes indicate parameters, which are lower in SOD1 G93A mice compared to wild type, and green box indicates parameters, which are higher in SOD1 G93A mice compared to wild type. Similarly, the color of the arrows indicates either a positive relationship (green) or a negative relationship (red), and their size indicates the relative strength of the relationship. The biology of these interactions is summarized below.

Mitochondria have a highly interactive dynamic with the endoplasmic reticulum (ER), the main intracellular calcium storehouse. Mitochondria take up calcium via the calcium-sensitive mitochondrial uniporter. However, sustained free cytosolic calcium inactivates the uniporter, preventing further calcium uptake (Moreau et al., 2006). Accumulated calcium in the mitochondria can then be released back into the cytosol via the sodium–calcium and hydrogen–calcium exchangers (Fuchs et al., 2013). Once intramitochondrial calcium rises above a certain level, the mitochondrial transition pore opens, initiating apoptotic or necrotic signaling cascades (Leung and Halestrap, 2008). Calcium originating from the activation of AMPA receptors and/or pathologically increased membrane permeability is thought to result in this shift of calcium from the ER to the mitochondria. Ryanodine receptors on the surface of the ER further amplify calcium-mediated calcium release from the ER, which in turn, could further exacerbate AMPA activation (Berridge, 2002). A second receptor that exacerbates calcium release from the ER is the calcium-activated IP3R.

Collectively, intracellular release of calcium from the ER, mitochondria, and other intracellular stores could explain the increase in intracellular cytosolic calcium concentration seen near onset (~100 days), which is mirrored by a paradoxical decrease in extracellular calcium entry (see **Figure 2C**). Another contributor for this apparent paradox could be a decrease in expression of calcium binders like calbindin D28K and parvalbumin (Celio, 1990), which have been proposed to result in increased cytosolic calcium in ALS mice (Appel et al., 2001). Chin et al. (2014) similarly shows a decrease in parvalbumin in SOD1 G93A mice as well as a reduction in sarcoplasmic/ER Calcium ATPase proteins, including SERCA1. Notably, calcium binders, which fall under the calcium sink category in this meta-analysis, show a slight dip that also corresponds to the timing of the intracellular calcium increase (**Figure 2C**).

Calcium release has a bi-directional relationship with ROS production since ROS homeostasis is maintained via Ca^2+^ signaling and Ca^2+^ dependent pathways. Calcium stimulates NO synthesis and leads to ROS production at Complex III (Feissner et al., 2009). Moreover, because the ryanodine receptor forms a tetramer with the sarcoplasmic and ERs, the reversible oxidation of endogenous superoxide groups can result in the release of additional calcium from the sarcoplasmic reticulum (Fill and Copello, 2002). Finally, oxidative agents like peroxide directly induce calcium release from the ER via IP3R (Wesson and Elliott, 1995). In summary, free radicals induce calcium leakage into the cytosol via the ryanodine receptor, Ca^2+^-leak channels, and inositol 1,4,5-trisphosphate receptors, and conversely, intracellular calcium concentration activates NOX and NOS, which then produces additional excess ROS and RNS, respectively (Chin et al., 2014). Ultimately, elevated internal calcium creates a cyclical feed forward mechanism that continually increases calcium and oxidative stress to the point of apoptosis (Chin et al., 2014).

Lower respiration rates, and consequently, lower intracellular ATP concentrations, directly contribute to the lowering of the mitochondrial potential, which can ultimately initiate apoptotic cascades. The impact of lower ATP concentration on oxidative and calcium imbalances is bi-directional, with increases in oxidants and calcium-mediated calcium release further impairing mitochondrial function, especially Complex 1, a key constituent for ATP production (Cassina et al., 2008; Cozzolino and Carri, 2012; Lautenschlager et al., 2013). Through a less direct path, an increase in oxidative stress can also lead to a swelling of mitochondria, which also further inhibits ATP production (Martin et al., 2007). Finally, lower concentrations of ATP impede calcium-ATPase in removing free calcium from the cytosol or shuttling calcium back to the ER for storage (Kaplan et al., 2003; Fuchs et al., 2013).

### Deciphering the Timing: Cause, Effect, and Instability

Because of their large size and innate emergent properties, motoneurons are susceptible to homeostatic instabilities. It has been previously shown that motoneurons, even in a physiological state, have insufficient mitochondrial capacity to buffer large calcium fluxes. Calcium buffering insufficiency is thought to be due to a reduced mitochondrial density per volume compared to non-motoneurons (Grosskreutz et al., 2007). Therefore, mitochondrial dysfunction and impaired calcium homeostasis is hypothesized to account for the selective vulnerability of motoneurons (Jaiswal and Keller, 2009; Jaiswal et al., 2009). Another contributor for the selective vulnerability of motoneruons is the requirement for axonal transport of mitochondria over very long distances, up to 1 m (Mitchell and Lee, 2009, 2012a; Lee and Mitchell, 2015). Finally, the dynamics of somatic input processing of motoneurons could explain the earlier death of fast twitch fibers in ALS (Mitchell and Lee, 2011).

Because there are so many interacting variables, it is difficult to determine which parameter(s) initiate versus simply affect the pathophysiological cyclical cascades of depressed cellular respiration, imbalances in calcium homeostasis, and intracellular elevation of oxidants in ALS. The presented data reveals that elevated oxidants appear later in the SOD1 G93A life span, closer to disease onset. However, both calcium and cellular respiration/mitochondrial mechanisms show early deficits. Much like the age-old question, “What came first, the chicken or the egg?” This meta-analysis begs the question, “What comes first—improper calcium homeostasis or depressed cellular respiration?”

Scientifically justified arguments could be made for either position. Increased calcium permeability or ATP depletion from sub-par cellular respiration, or a combination of both, could initiate a dynamic instability in the motoneuron that results in the ALS phenotype. The trend lines presented in this meta-analysis reveal the presence of potential compensatory mechanisms, which attempt but ultimately fail, to re-stabilize to homeostasis. For example, between 0 and 20 days, there is a rise in HSPs and a gradual increase in calcium sensitivity. The “slight bump” in mitochondrial mechanisms/cellular respiration at pre-onset also coincides with the lowest intracellular calcium levels. Attempts to re-stabilize to homeostasis could potentially correspond to the small fluctuations apparent in **Figures 1** and **2**, although more data is needed to definitively determine their statistical significance.

Mathematical instabilities within pathophysiological feedback loops have already been identified in a dynamic meta-analysis of the SOD1 G93A mouse model (Mitchell and Lee, 2012b). If unstable pathology dynamics are the actual underlying culprit, it may not actually matter exactly which mechanism first initiated the cascade (see Future Directions). Both preclinical and clinical failures to obtain meaningful success using single-mechanism treatments illustrate the potential validity of this point. Among the many examples are: dichloroacetate (Miquel et al., 2012), which attempts at restoring the mitochondrial respiratory capacity in the astrocytes, *N*-acetyl-glucagon-like peptide-1 (Sun et al., 2013), which endogenously regulates metabolism by promoting insulin synthesis and secretion, and creatine (Groeneveld et al., 2003; Snow et al., 2003; Beal, 2011), which is known to enhance ATP synthesis. However, no single treatment to targeting cellular energetics has been effective enough to translate to an effective treatment for humans (Tadic et al., 2014).

In this meta-analysis, we reveal that, although there are some small fluctuations, cellular respiration is depressed for the entire SOD1 G93A ALS mouse lifespan. Interestingly, ALS patients, prior to the onset of their ALS, have been found to be healthier (e.g., less antecedent disease) than age, gender, and geography-matched control subjects (Mitchell et al., 2015). However, such patients could still have asymptomatic pre-ALS variations in their underlying motoneuron regulation, which make them more susceptible to instabilities. Hypervigilant regulation as been put forth as one possibility to explain how the above-average pre-ALS health of patients could be correlated to a later, destabilizing motoneuron perturbation, which initiates ALS (Mitchell et al., 2015). “Hypervigilant regulation” results when underlying regulatory processes aggressively overreact to correct imbalances from homeostasis, making them ‘hypervigilant’ to perturbation (in control theory, referred to as a too-high feedback gain). While hypervigilant regulation would initially be overall protective, it could also result in greater later susceptibility to destabilization, especially in highly susceptible motoneurons (Mitchell et al., 2015). The temporal calcium, oxidant, and HSP fluctuations identified in this study, in combination with the oscillatory behavior of other previously identified parameters (Mitchell and Lee, 2012b) such as axonal transport (Mitchell and Lee, 2012a) and excitability (Delestree et al., 2014), are suggestive of the possible role of regulatory and homeostatic impairments as being the “cause” of ALS.

### Future Directions

Perhaps instead of focusing on mechanistic initiation, treatments should focus on treating the underlying instability, itself (Mitchell and Lee, 2008, 2012b). Whether in engineering process or in biology, treating instability typically requires impacting multiple targets or feedback loops, which may or may not have directly initiated the destabilizing perturbation or event. Combination treatments can leverage synergistic interactions to increase treatment effect size. Multiple experimentalists have attempted combinatory treatments on the SOD1 G93A mouse model (Waibel et al., 2004; Feng et al., 2008; Del Signore et al., 2009; Del Barco et al., 2011). For example, Waibel et al. (2004) experimented with rasagiline, an anti-apoptotic with neuroprotective properties, combined with riluzole, a sodium channel blocker, to reduce excitotoxicity. The combinatory treatment did exhibit a statistically significant improvement compared to control and compared to Riluzole alone.

In addition to exploiting synergistic interactions to increase effect size, combination treatments could potentially be used to re-stabilize the system to homeostasis. Theoretical SOD1 G93A ALS models of combination treatments have shown this exciting possibility (Mitchell and Lee, 2012b). In fact, of the several thousand computationally assessed combination treatment permutations, a few percent of 2 and 3-way treatment strategies were able to mathematically stabilize the ALS pathophysiology (Mitchell and Lee, 2012b). Interestingly, energetics was one of the pathophysiological categories that most frequently appeared in synergistic or stabilizing treatment combinations. Given the early and lasting depression of ATP and respiration rates identified in the present study, it is not surprising that energetics was previously predicted to have the greatest single-category effect size (Mitchell and Lee, 2012b).

Based on the results of the present study, it would appear that therapeutics leveraging the strong interactions within the calcium-respiration-oxidation triad could be promising. As shown in **Figures 1** and **2**, prior to onset, it appears the SOD1 G93A physiology temporarily compensates toward decreasing intracellular calcium, increasing anti-oxidative HSPs, and slightly increasing respiration rate; thus, treatment to amplify these existing compensatory mechanisms would seem intuitive. However, like spinal cord injury (Mitchell and Lee, 2008), such treatments would likely have to be initiated very early in the disease process to have a meaningful effect. In fact, in the case of instability, the timing of treatment may be the most important parameter, especially given human patients will not be treated until after ALS symptoms appear. Obtaining a finer point on the timing and statistical significance of fluctuations in intracellular calcium, ATP concentration, and free radicals, is critical to devising combination treatments that have clinically significant results. An additional essential research path is better assessment of homeostatic regulation. Modulation of regulatory pathways may prove more fruitful for re-stabilization than direct physical or chemical manipulation of cellular elements.

## Conflict of Interest Statement

The authors declare that the research was conducted in the absence of any commercial or financial relationships that could be construed as a potential conflict of interest.
